# Hypothalamic *agrp* and *pomc* mRNA Responses to Gastrointestinal Fullness and Fasting in Atlantic Salmon (*Salmo salar*, L.)

**DOI:** 10.3389/fphys.2020.00061

**Published:** 2020-02-11

**Authors:** Tharmini Kalananthan, Koji Murashita, Ivar Rønnestad, Mitsumasa Ishigaki, Kota Takahashi, Marta S. Silva, Yuki Wakabayashi, Floriana Lai, Munetaka Shimizu, Tom O. Nilsen, Enrique Pino Martinez, Ana S. Gomes

**Affiliations:** ^1^Department of Biological Sciences, University of Bergen, Bergen, Norway; ^2^Research Center for Aquaculture Systems, National Research Institute of Aquaculture, Japan Fisheries Research and Education Agency, Tamaki, Japan; ^3^Department of Animal Sciences, Nagoya University, Nagoya, Japan; ^4^Department of Biology, Miyagi University of Education, Sendai, Japan; ^5^Institute of Marine Research, Bergen, Norway; ^6^Faculty of Fisheries Sciences, Hokkaido University, Hakodate, Japan; ^7^Norwegian Research Center, NORCE Environment, Bergen, Norway

**Keywords:** Atlantic salmon, hypothalamus, *agrp*, *pomc*, fullness, fasting, gastrointestinal tract

## Abstract

The orexigenic agouti-related protein (AgRP) and the anorexigenic pro-opiomelanocortin (POMC) are crucial players in the control of feed intake in vertebrates, yet their role in teleosts has not been fully established. Triplicate groups of Atlantic salmon (*Salmo salar*) post smolts were subjected to (1) fasting for 3 days (fast) and (2) normal feeding (fed), resulting in a significant (*p* < 0.05) upregulation of hypothalamic *agrp1* transcripts levels in the fast group. Moreover, the mRNA abundance of *agrp1* was significantly (*p* < 0.05) correlated with the stomach dry weight content. Corresponding inverse patterns were observed for *pomca2*, albeit not statistically significant. No significant differences were found for the other paralogues, *agrp2* and *pomca1* and *b,* between fed and fast groups. The significant correlation between stomach fullness and *agrp1* mRNA expression suggests a possible link between the stomach filling/distension and satiety signals. Our study indicates that hypothalamic *agrp1* acts as an orexigenic signal in Atlantic salmon.

## Introduction

Food intake and appetite are controlled by the integration of peripheral and central signals in the hypothalamus of vertebrates (Volkoff, 2016; Delgado et al., 2017; Rønnestad et al., 2017). Appetite-stimulating (orexigenic) and appetite-inhibiting (anorexigenic) factors are key drivers of feeding, and several studies have suggested that their functional role have been evolutionary conserved across vertebrates, including teleosts (Volkoff, 2016; Rønnestad et al., 2017; Soengas et al., 2018). In many teleost species, two agouti-related protein (*agrp*) paralogous genes (*agrp1* and *agrp2*) have been identified (Agulleiro et al., 2014; Shainer et al., 2019), including in Atlantic salmon (*Salmo salar*) (Murashita et al., 2009a). In addition, the orexigenic role of hypothalamic AgRP appears to be conserved in some of the teleost species studied, such as goldfish (*Carassius auratus*) (Cerda-Reverter and Peter, 2003), zebrafish (*Danio rerio*) (Song et al., 2003; Shainer et al., 2019), coho salmon (*Oncorhynchus kisutch*) (Kim et al., 2015), and gilthead seabream (*Sparus aurata*) (Koch et al., 2018). However, in Atlantic salmon, previous results indicated that *agrp1* may have an anorexigenic effect based on analyses of the whole brain mRNA expression after 6 days of fasting, while *agrp2* had no effect on the control of appetite (Murashita et al., 2009a).

Proopiomelanocortin (POMC) is a precursor peptide which is post-transcriptionally cleaved into melanocyte-stimulating hormones (α-, β- and γ-MSH) and the adrenocorticotropic hormone (ACTH) (Castro and Morrison, 1997). In Atlantic salmon, three *pomc* paralogous genes (*pomca1*, *pomca2*, and *pomcb*) and one splice variant (*pomca2s*) have been previously identified and characterized (Murashita et al., 2011). In mammals, MSHs are involved in appetite control (Saneyasu et al., 2011), while in teleosts their functional role in appetite control remains to be clarified. For example, fasting did not change *pomc* expression in goldfish (Cerdá-Reverter et al., 2003), but intracerebroventricular administration of α-MSH showed an anorexigenic effect for this species (Matsuda et al., 2008; Kojima et al., 2010). In zebrafish, a cyprinid species as goldfish, it has been shown that *pomca* expression decreased in starved larvae (Liu et al., 2016). In salmonids, fasting has triggered a decrease in *pomca1* (but not *pomca2* or *pomcb*) expression in the hypothalamus of rainbow trout (Leder and Silverstein, 2006) and in whole brain of Atlantic salmon (Valen et al., 2011). These results are consistent with the anorexigenic role reported for mammals.

Signals from the gastrointestinal tract, such as sense of *fullness*, are important for appetite control and contribute to regulate food intake on a meal-to-meal basis (Sam et al., 2012). After a meal, the distension of the stomach and interactions between nutrients and the gut wall trigger the secretion of several peptide hormones, communicating the filling along with luminal nutrient status to the hypothalamus. This applies also to salmonids, as satiety signals from the gastrointestinal tract have a major impact on appetite (Grove et al., 1978). In rainbow trout, for instance, appetite returned (i.e., fish restarted feeding) when 80–90% of the stomach content from the previous meal was transferred downstream into the proximal gut (Ware, 1972).

In the Atlantic salmon aquaculture production, a period of fasting that lasts for 2–4 days is a common practice prior to handling, transportation, and harvest (Waagbø et al., 2017). This practice allows complete evacuation of the gut and an empty digestive tract, which minimizes impacts on fish welfare and ensures proper hygiene after harvest (Einen et al., 1998; Robb, 2008). Fasting also suppresses any postprandial elevation of metabolic rate, thereby permitting the fish to allocate more energy towards swimming and stress handling (Waagbø et al., 2017). Uncovering the impacts of these fasting periods on appetite and food intake control is therefore essential to optimizing their recovery. In this study, we investigated the effect of 3 days of fasting on hypothalamic *agrp* (*1* and *2*) and *pomc* (*a1, a2* and *b*) and explored the relationship between appetite and gastrointestinal filling.

## Materials and Methods

### Ethical Treatment of Animals

The research and sampling were conducted in accordance with the Norwegian Animal Research Authority regulations and was approved by the local representative of Animal Welfare at the Department of Biological Sciences, University of Bergen (Norway).

### Experimental Setup and Sampling

We obtained Atlantic salmon individuals from Bremnes Seashore’s RAS facility (Trovåg, Norway). Fish were randomly assigned to tanks (5 fish per tank) and acclimatized to the experimental setting consisting of six freshwater indoor 150 L tanks and water temperature at 8.5°C. Continuous day light was used to mimic the standard commercial procedures and to stimulate optimal growth (Hansen et al., 1992). During the 18 days of acclimation period, all tanks were fed daily *ad libitum* from 9:00 to 16:00 h with commercial dry fish pellets (Biomar 3 mm) using automatic fish feeders.

To evaluate the effect of the fasting, 14 Atlantic salmon post smolts (average body weight 214.7 ± 61.7 g and length 26.8 ± 2.4 cm) were sampled from two groups that were either fed (sampled 2 h after feeding) or fasted for 3 days. In total, seven fish per group were sampled (2 or 3 fish per tank). Atlantic salmon were euthanized with a lethal dose of 200 mg/l of MS222 (Tricaine methanesulfonate, Sigma-Aldrich, MO, USA). The whole brain was removed from the skull, and the hypothalamus sampled and stored in RNAlater (Thermo Fisher Scientific, MA, USA). The Fulton’s condition factor (*K*) was determined at the sampling time using the equation:

$$K=100\left(\;\frac{W}{L^{3}}\right)$$

where, *W* is the weight (g) and *L* is the length of the fish (cm) (Froese, 2006).

### Gastrointestinal Tract Compartments Filling

We dissected and carefully divided the gastrointestinal tract into three compartments (see Supplementary Figure 1A): stomach (ST), midgut (MG), and hindgut (HG), using surgical clamps to avoid loss or transfer of content between compartments. Next, each segment was emptied of food and digesta by gently stroking the content out onto pre-weighed pieces of aluminium foil. The weight of contents in each segment was first measured on a wet weight basis, and thereafter, dry weight was obtained after incubating in an oven at 110°C for at least 3 h, until it was completely dried.

### mRNA Abundance Analysis by RT-qPCR

Total RNA was isolated from the hypothalamus using TRI reagent (Sigma-Aldrich) according to the manufacturer’s instructions. Samples were treated with TURBO DNA-free (Thermo Fisher Scientific) to eliminate possible genomic DNA contamination. Quality of DNase treated total RNA was assessed on all samples using an Agilent 2100 Bioanalyzer (Agilent Technologies, CA, USA). All samples had a RNA integrity number (RIN) equal or higher than 9 (scale 1–10). cDNA was synthesized from 1.0 μg of DNase treated total RNA using oligo (dT) primer from SuperScript III First-Strand Synthesis system for RT-PCR kit (Thermo Fisher Scientific).

Specific primers spanning an exon-exon junction were designed for all the target genes (Table 1). qPCR reactions were performed in duplicates using iTaq Universal SYBR Green Supermix (Bio-Rad, CA, USA) in a 20 μl final reaction volume. The qPCR reactions were performed in a Bio-Rad CFX96™ Real-Time System with the following cycling conditions: 95°C for 30 s; 40 cycles of 95°C for 5 s, 60°C for 25 s. Melting curve analysis over a range of 65–95°C (increment of 0.5°C for 2 s) allowed for the detection of possible nonspecific products and/or primer dimers.

**Table 1:** Sequence of the specific primers used for qPCR mRNA expression analysis. Primer sequences, amplicon sizes, *R*^2^, and qPCR efficiency are indicated for each primer pair.

Gene	GenBank acc. no.	Sequence (5′ → 3′)	Amplicon (bp)	*R*^2^	Efficiency (%)
*agrp1*	NM_001146677.1	F: ATGGTCATCTCAGTATTCCCAT	152	0.9995	93
		R: AGAGAGCCTTTACCGATATCTG			
*agrp2*	NM_001146678.1	F: TGTTTCGCCGAAGACCTGAA	142	0.9997	98
		R: GTTTCTGAAATGCAACGTGGTG			
*pomca1*	NM_001198575.1	F: ATACTTTTGAAACAGCGTGACGA	108	0.9997	101
		R: CAACGAGGATTCTCCCAGCA			
*pomca2*	NM_001198576.1	F: TTTGGCGACAGGCGAAGATG	91	0.9949	94
		R: TCCCAGCACTGACCTTTCAC			
*pomcb*	NM_001128604.1	F: CAGAGGACAAGATCCTGGAGTG	182	0.9916	103
		R: TTTGTCGCTGTGGGACTCAG			

Standard curves relating initial template quantity to amplification cycle were generated from the target gene cloned into pCR4-TOPO vector (Thermo Fisher Scientific) using a 10-fold stepwise dilution series. The standard curves were used to determine the qPCR efficiency for each assay (Table 1). The copy number was determined for each gene/sample based on the respective standard curve, using the following equation:

$$\mathrm{Copy}\;\mathrm{number}=10^{\left(\frac{Cq-\mathrm{Intercept}}{\mathrm{slope}}\right)}$$

### Statistical Analysis

**Figure 1:**
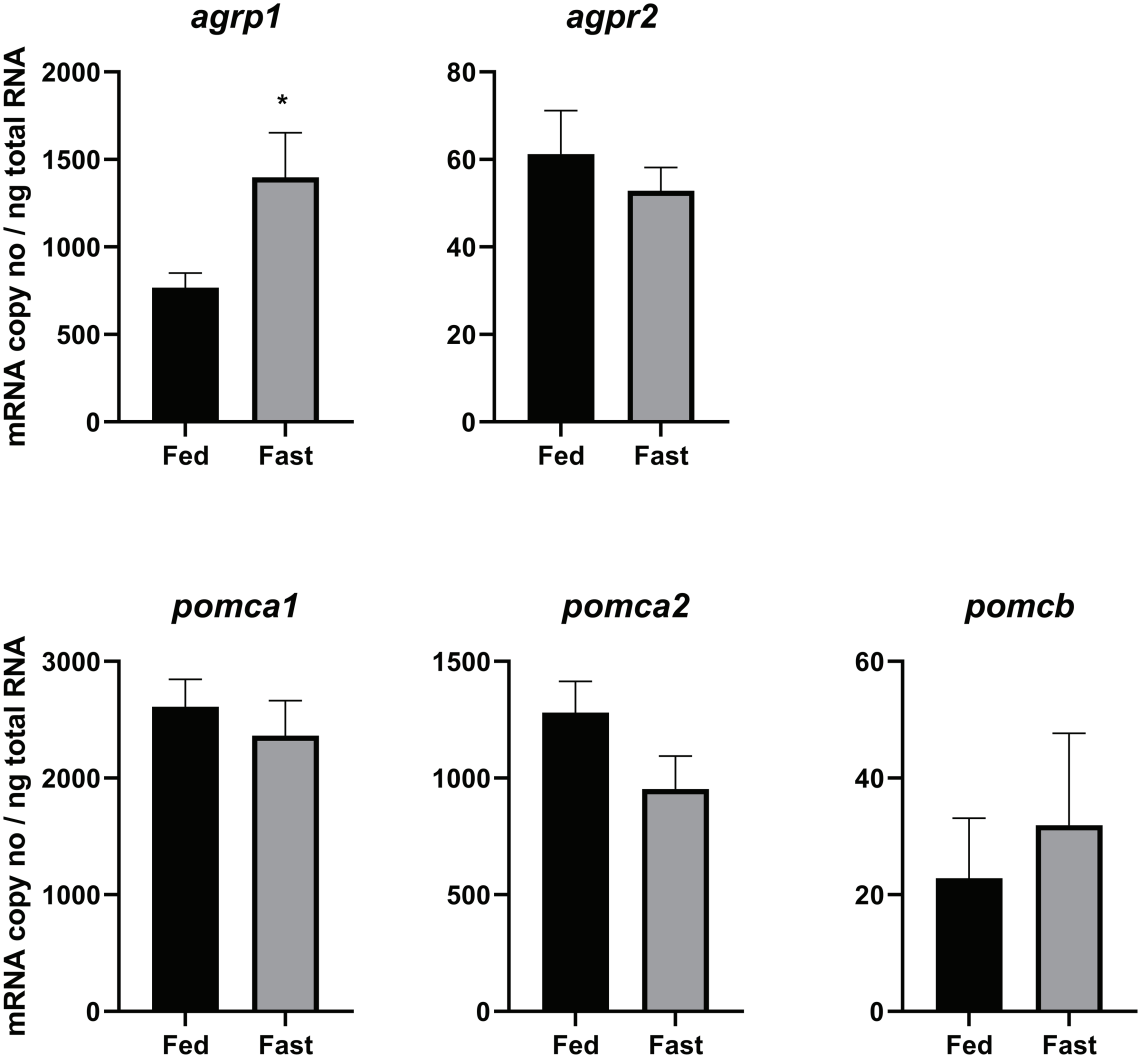
Effects of 3 days of fasting on hypothalamic mRNA expression of *agrp1*, *agrp2*, *pomca1*, *pomca2*, and *pomcb*. Results are presented as mean ± SEM (*n* = 7). Asterisk (*) indicates statistically significant difference (*p* = 0.02). For detailed statistical information, refer to Supplementary Table 1.

All mRNA expression data were tested for normality using Shapiro-Wilk W-test and subsequently log-transformed to ensure that it followed a normal distribution. Correlation analyses were conducted with generalized linear models (GLM) assuming a normal distribution. The effects of treatment (fed versus fast) on condition factor (*K*) and on the mRNA expression levels (copy numbers of each transcript) were evaluated (*n* = 7 fish per treatment group). It was also tested the effects of gastrointestinal dry weight contents (ST, MG, and HG) on the mRNA levels of each transcript. In addition, it was assessed the relationship between gastrointestinal dry weight contents and wet weight and tested for tank effects on mRNA expression levels by adding the tank as an interaction factor. All statistical analyses were carried out by RCoreTeam (2018), using the package ggplot (Wickham, 2016) to plot graphs. The plot bar graphs (Figure 1; Supplementary Figure 1) were produced in GraphPad Prism version 8.2.0 for Windows (GraphPad Software, CA, USA). Statistical significance was considered at *p* < 0.05.

## Results

### Gastrointestinal Tract Fullness and Condition Factor (*K*)

The condition factor significantly (*p* = 0.01) decreased in the fast group (1.06 ± 0.03) compared to the fed group (1.11 ± 0.02). The content of the three different regions of the gastrointestinal tract (ST, MG, and HG) was significantly different between the fed and fast group (see Supplementary Figure 1). In fact, the fast group had no ST content, and both MG and HG content was much lower than in the fed group. The wet and dry content weight was highly correlated in all the three gut sections (Supplementary Figure 2).

### Effects of Fasting on *agrp* and *pomc* mRNA Levels and Correlation With Gut Sections Fullness

**Figure 2:**
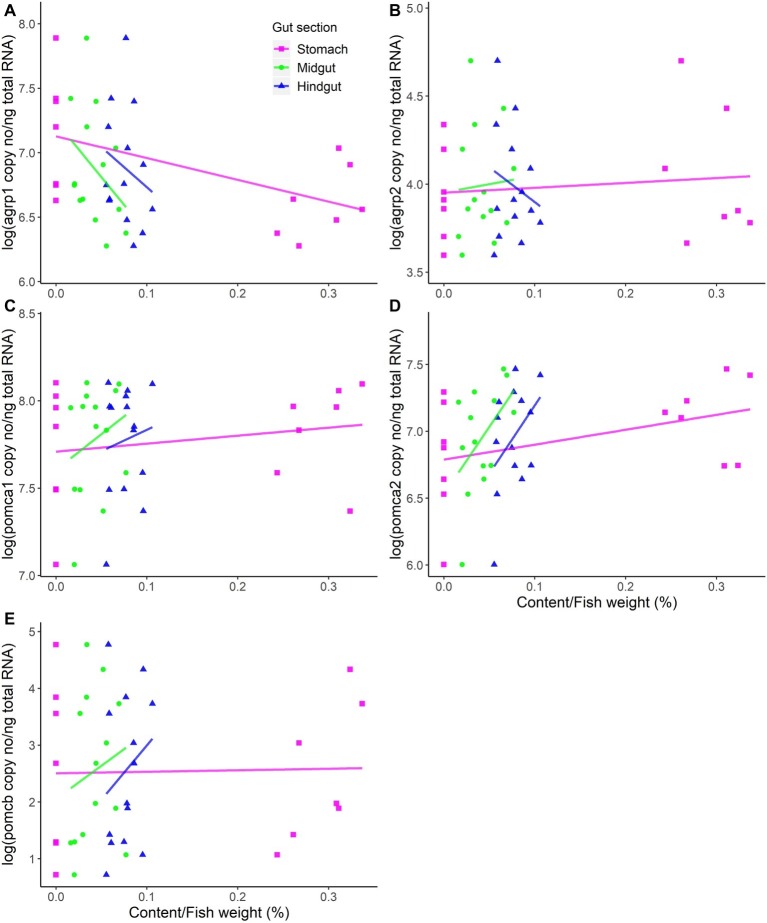
mRNA expression levels versus content in the different compartments of the gastrointestinal tract (stomach, midgut, and hindgut). **(A)** *agrp1*, **(B)** *agrp2*, **(C)** *pomca1*, **(D)** *pomca2*, and **(E)** *pomcb* mRNA expression is presented as the log-transformed copy number (no) per nanograms (ng) of total RNA. Dots represent all individual fish (*n* = 14), while solid lines represent linear regressions estimated by the general linear model (GLMs). The weight of gastrointestinal tract content was standardized by the fish weight. For detailed statistical information, refer to Supplementary Table 2.

Fasting significantly (*p* = 0.02) upregulated hypothalamic *agrp1* mRNA expression (Figure 1). Furthermore, *pomca2* showed an opposite trend and appeared to be downregulated by fasting; however, the differences were not statistically significant (Supplementary Table 1). No other significant differences were observed for *agrp2*, *pomca1*, and *pomcb* between fed and fast Atlantic salmon (Figure 1). Moreover, *agrp1* mRNA levels and stomach filling were significantly (*p* = 0.03) correlated (Figure 2A; Supplementary Table 2), while no other statistically significant correlation was found between *agrp2*, *pomca1*, *pomca2*, and *pomcb* mRNA copy number and gastrointestinal compartments content (Figures 2B–E).

There were no statistically significant effects of the tanks on the expression levels of the target genes.

## Discussion

The present study adds to and partially revises the existing knowledge on neuroendocrine control of appetite in Atlantic salmon, which was based on analysis of whole brain instead of specific brain regions. Here, we focused on the hypothalamus that is considered the hub for the appetite control in vertebrates. As in mammals, the teleost feeding center seems to reside in the hypothalamic area (reviewed in Peter (1979)). Furthermore, several neuropeptides involved in appetite control, including AgRP and POMC, are present in the hypothalamus of teleost species (Cerdá-Reverter et al., 2003; Cerda-Reverter and Peter, 2003; Otero-Rodiño et al., 2019). Different nutrient status also modulate the expression of teleost hypothalamic neuropeptides (reviewed in Volkoff (2016); Delgado et al. (2017); Rønnestad et al. (2017)). However, we cannot rule out that other areas of the brain might act as feeding centers in teleost fishes, as reviewed in Cerdá-Reverter and Canosa (2009) and Soengas et al. (2018), emphasizing the importance to explore the role of each brain region in appetite control.

Our results show that 3 days of fasting significantly increased hypothalamic *agrp1* mRNA expression, suggesting that *agrp1* acts as an orexigenic factor in Atlantic salmon. This contrasts previous findings for this species (Murashita et al., 2009a), providing novel insights that may revise the current knowledge on orexigenic and anorexigenic factors in salmon. Our data are in agreement with the suggested AgRP orexigenic role for other vertebrates including several fish species, such as goldfish (Cerda-Reverter and Peter, 2003), zebrafish (Song et al., 2003; Jeong et al., 2018; Shainer et al., 2019), seabass (*Dicentrarchus labrax*) (Agulleiro et al., 2014), Ya fish (*Schizothorax prenanti*) (Wei et al., 2013), arctic char (*Salvelinus alpinus*) (Striberny et al., 2015), and coho salmon (Kim et al., 2015). The discrepancies observed between the results obtained in the present study and the previous studies from Valen et al. (2011), which indicated that *agrp1* have an anorexigenic role, are most likely a result from hypothalamus versus whole brain analysis. It is possible that *agrp1* is also abundant in non-hypothalamic regions of the brain (Kurokawa et al., 2006) and offers other functional roles than appetite control (Xiao et al., 2003). This denotes the importance of analyzing individual tissues/organs and the need to revisit previous data in a more detailed manner. However, we cannot rule out the hypothesis that the controversial findings may be a consequence of the different sampling times, i.e., 3 days fasting in the present study versus 6 days (Murashita et al., 2009a) or 30 min to 24 h (Valen et al., 2011) fasting periods. Therefore, a future detailed study including several sampling time points will be necessary to ascertain the current hypothesis of this study. Furthermore, *in situ* hybridization and immunohistochemistry studies will be essential to reveal the location of AgRP1 expression within the hypothalamus and investigate its possible co-localization with other neuropeptides, such as neuropeptide Y (NPY), POMC or cocaine- and amphetamine-regulated transcript (CART).

We found a correlation between *agrp1* mRNA levels and stomach filling content, which may support the hypothesis that *agrp1* is also an important orexigenic factor in Atlantic salmon. Previous studies in rainbow trout (150–200 g) have shown that appetite and fullness had an almost perfectly inverse relationship, with appetite return reaching its maximum level when fullness approaches 0 and vice-versa (Grove et al., 1978). Furthermore, ca. 50 h for a complete gastric emptying in fish with a 150–250 g of weight was indicated. Our study is in line with these results, as 3 days of fasting resulted in a complete empty stomach and increased levels of the orexigenic *agrp1*, supporting the hypothesis proposed by Grove et al. (1978) that the return of appetite is proportional with the emptying of the stomach. In addition, it has been previously reported by Murashita et al. (2009b) that 6 days of fasting induced upregulation of stomach *ghrelin 1* in Atlantic salmon. The orexigenic hormone ghrelin promotes the release of hypothalamic AgRP (reviewed in Nuzzaci et al., 2015). Therefore, we can hypothesize that also in Atlantic salmon fasting induces the increase of ghrelin levels, which, consequently triggers the increase expression of *agrp1* in the hypothalamus. However, this hypothesis needs to be further investigated, particularly because the current study is limited to only two very distinct phases, i.e., full versus empty stomach, and therefore other factors, such as nutritional conditions, may also contribute to the hypothalamic expression of the neuropeptides analyzed. For example, in mammals, it has been shown that increased glucose levels inhibit the NPY/AgRP neurons (reviewed in Marty et al., 2007); however, it seems hypothalamic *agrp* mRNA abundance is not affected by hyperglycaemic treatment in rainbow trout (Otero-Rodiño et al., 2015). The link between appetite (mRNA expression of orexigenic/anorexigenic factors) and stomach fullness, including peripheral hormones, and/or the role of the nutritional status are important issues that require further research.

The analysis of expression of *agrp2* revealed that fasting has no effect on its mRNA levels, confirming the observations by Murashita et al. (2009a). In addition, the hypothalamic *agrp2* expression levels were much lower than the levels of *agrp1* (Figure 1). All together, these results suggest that Atlantic salmon *agrp2* may have other functional roles than controlling appetite. This would correspond to findings in zebrafish (Shainer et al., 2017) suggesting that pineal *agrp2* regulates background pigment adaptation for camouflage (Zhang et al., 2010) and pre-optic *agrp2* acts as a neuroendocrine modulator of the stress response (Shainer et al., 2019).

Among the Atlantic salmon *pomc* genes analyzed in this study, only *pomca2* appears to be possibly downregulated after 3 days of fasting, opposite to previous findings in Atlantic salmon whole brain analysis after 6 days of fasting (Valen et al., 2011) where only *pomca1* significantly decreased after fasting. The anorexigenic role of *pomc* have been reported for several fish species (reviewed in Volkoff, 2016), including salmonids, for e.g., in coho salmon intra-peritoneal injections of α-MSH suppressed feed intake (White et al., 2016). However, conflicting data have also been reported for other salmonids: in rainbow trout 14 days of fasting did not affect *pomc* transcripts expression levels, whereas 28 days of fasting resulted in a significant decrease of hypothalamic *pomca1* but not for *pomca2* or *pomcb* (Leder and Silverstein, 2006), and 4 months of fasting (118 days) resulted in a significant increase of both hypothalamic *pomca1* and *pomcb* expression levels. The very low mRNA expression levels here reported for hypothalamic *pomcb* suggests that this gene may not serve as an appetite-controlling factor in the hypothalamus of Atlantic salmon. This hypothesis can be also supported by the fact that intraperitoneal deliver of leptin, a strong anorexigenic hormone, did not affect *pomcb* mRNA expression in Atlantic salmon (Murashita et al., 2011) or in rainbow trout (Murashita et al., 2008). Taken together, it can be hypothesized that hypothalamic *pomca2* and possibly *pomca1* (Valen et al., 2011), but not *pomcb* function as an anorexigenic factor in Atlantic salmon; however, this needs to be further investigated.

In summary, our study demonstrates for the first time a correlation between an appetite related neuropeptide, hypothalamic *agrp1*, and stomach filling in a teleost species. Three days of fasting upregulated hypothalamic *agrp1* mRNA expression levels, suggesting an orexigenic role of this gene in Atlantic salmon and indicating a different role in appetite control than the one proposed for whole brain *agrp1* by Murashita et al. (2009a) and Valen et al. (2011). The *agrp1* response observed in this study suggests that this gene plays a role in the control of appetite in Atlantic salmon, enabling the fish to cope with short-term fasting periods and recovery after fasting. Our study provides a basis to form hypotheses about the differential expression patterns of appetite-controlling factors that need to be further explored. Further research needs to focus on tissue-specific analysis in Atlantic salmon, including central and peripheral signals, and how their interaction affects fish health and welfare during sensitive production stages, such as those requiring short-term fasting periods.

## Data Availability Statement

All datasets generated for this study are included in the article/Supplementary Material.

## Ethics Statement

The experiment was conducted in an experimental facility approved to conduct experiments with teleosts and in accordance with the rules and regulations of the Norwegian Animal Research authority. The trial mimicked commonly used practices in the aquaculture industry, including 3–4 days of fasting prior transportation, handling, and harvest. The protocol used was approved by the local representative for animal welfare at the Department of Biological Sciences, University of Bergen (Norway). The responsible senior faculty members responsible for sampling were all accredited by Federation of European Laboratory Animal Science Associations (FELASA).

## Author’s Note

This work is a result of an educational Summer School student project hosted by University of Bergen from 17 to 28 June, 2019.

## Author Contributions

KM, IR, and AG designed the study. KM, MI, KT, MSi, YW, and AG performed the laboratory analysis. All authors contributed to the sampling and data analysis, writing of the manuscript, and approved the final version.

### Conflict of Interest

The authors declare that the research was conducted in the absence of any commercial or financial relationships that could be construed as a potential conflict of interest.
