# Supplementary material for: Hypothalamic agrp and pomc mRNA Responses to Gastrointestinal Fullness and Fasting in Atlantic Salmon (Salmo salar, L.)
Source: Front Physiol. 2020 Feb 11;11:61. doi: 10.3389/fphys.2020.00061 (PMC7026680; doi:10.3389/fphys.2020.00061)
Supplement: Supplementary file 1 [file Table_1.docx]

Supplementary Material

## Supplementary Figures


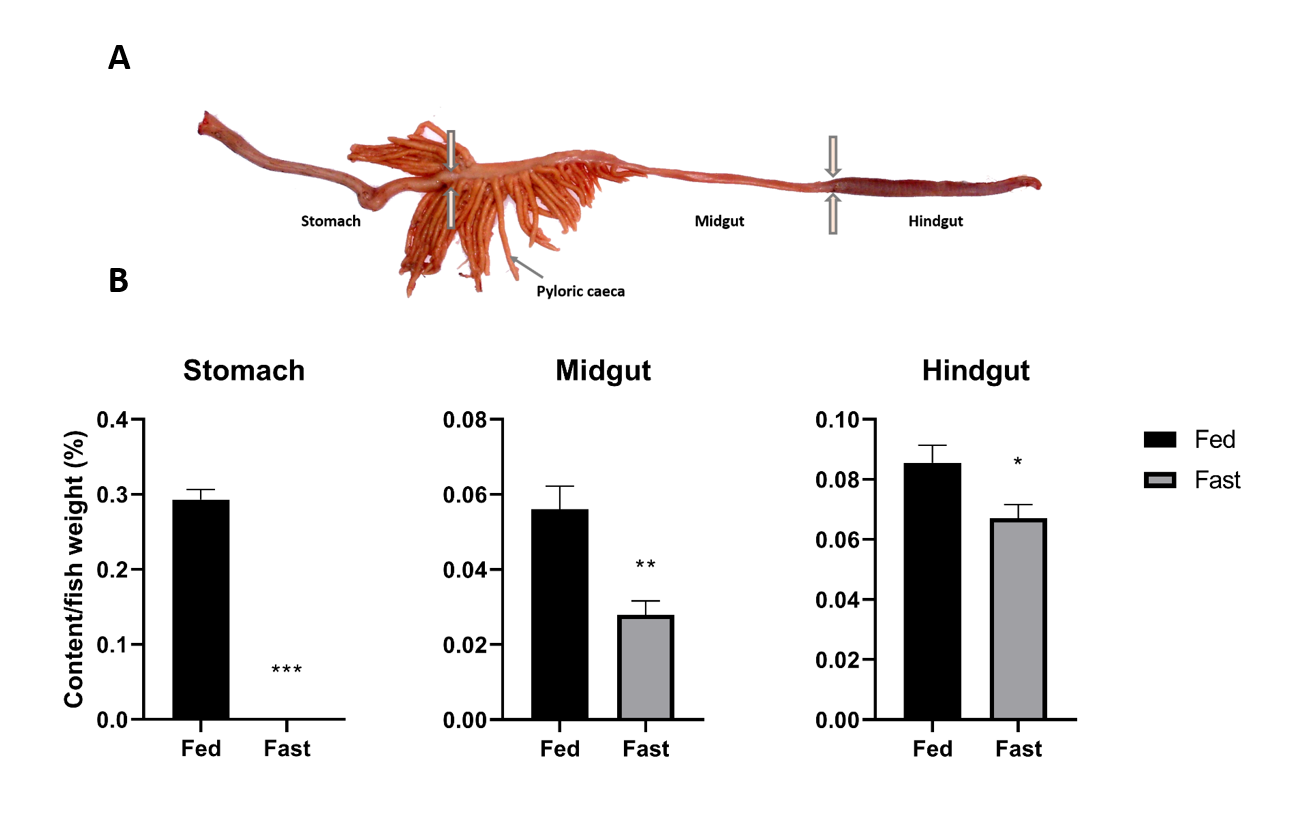
**Supplementary Figure 1.** **A)** Picture of the Atlantic salmon gastrointestinal tract. Arrows represent the positions where surgical clamps were used to divide the gut into three distinct compartments. Photo by Dr. Koji Murashita; **B)** Stomach, midgut and hindgut content dry weight (g) standardized by the fish weight. Comparison between fed (black bars) and fast (grey bars) fish. Results are presented as mean ± SEM (*n*=7). Asterisks indicate statistically significant differences: *** p < 0.001; ** p <0.01; * p < 0.05.

**
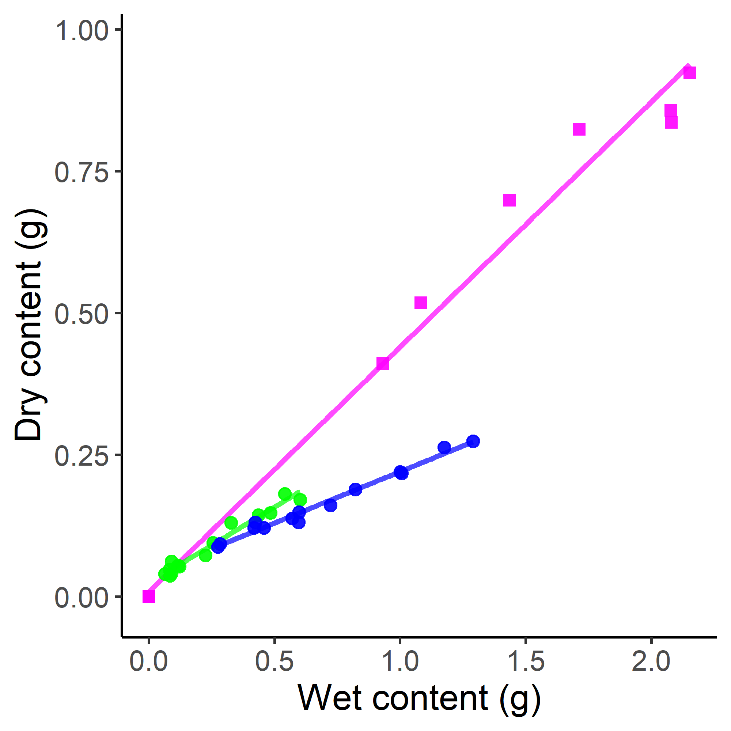
**

**Supplementary Figure 2:** Correlation between dry weight (g) and wet weight (g) content for stomach (magenta), midgut (green) and hindgut (blue). Dots represent all individual fish (n = 14), while solid lines represent linear regressions estimated by the general linear model (GLMs). The statistically significant differences were as follows: stomach *p* = 2.28e-13; midgut *p* = 3.6e-10; hindgut *p* = 5.19e-12. The 95% confidence interval is as follows: ± 0.13 for stomach; ± 0.38 for midgut; and ± 0.40 for hindgut.

## Supplementary Tables

**Supplementary table 1:** Results from the generalized linear model for log transformed mRNA expression analysis between fed and fast groups (*n* = 7 per group). Estimates, 95% confidence interval and ***p-***values are given.

| **Gene** | **Estimate** | **95% confidence interval** | ***p*-value** |
| --- | --- | --- | --- |
| *agrp1* | -0.54 | ± 0.39 | 0.02 |
| *agrp2* | 0.11 | ± 0.35 | 0.54 |
| *pomca1* | 0.13 | ± 0.33 | 0.48 |
| *pomca2* | 0.34 | ± 0.39 | 0.12 |
| *pomcb* | -0.1 | ± 1.45 | 0.9 |

**Supplementary table 2:** Results from the generalized linear model for log transformed mRNA expression levels *versus* gastrointestinal tract dry weight content (*n* = 7 per group). Estimates, 95% confidence interval and ***p-***values are given.

| **Gene** | **Gut compartment** | **Estimate** | **95% confidence interval** | ***p*-value** |
| --- | --- | --- | --- | --- |
| *agrp1* | Stomach | -1.69 | ± 1.39 | 0.03 |
|  | Midgut | -8.65 | ± 12.41 | 0.20 |
|  | Hindgut | -6.22 | ± 15.52 | 0.45 |
| *agrp2* | Stomach | 0.28 | ± 1.18 | 0.65 |
|  | Midgut | 0.95 | ± 9.29 | 0.84 |
|  | Hindgut | -3.95 | ± 10.88 | 0.49 |
| *pomca1* | Stomach | 0.46 | ± 1.16 | 0.45 |
|  | Midgut | 4.10 | ± 9.02 | 0.39 |
|  | Hindgut | 2.45 | ± 11.03 | 0.67 |
| *pomca2* | Stomach | 1.12 | ± 1.33 | 0.12 |
|  | Midgut | 10.09 | ± 10.09 | 0.07 |
|  | Hindgut | 10.22 | ± 12.58 | 0.14 |
| *pomcb* | Stomach | 0.27 | ± 4.90 | 0.92 |
|  | Midgut | 11.75 | ± 38.24 | 0.56 |
|  | Hindgut | 19.11 | ± 45.04 | 0.42 |
